# Sustained effects of corrupted feedback on perceptual inference

**DOI:** 10.1038/s41598-019-41954-z

**Published:** 2019-04-02

**Authors:** R. S. Varrier, H. Stuke, M. Guggenmos, P. Sterzer

**Affiliations:** 1 Department of Psychiatry and Psychotherapy, Charité – Universitätsmedizin Berlin, corporate member of Freie Universität Berlin, Humboldt-Universität zu Berlin, and the Berlin Institute of Health, Berlin, Germany; 2 Bernstein Center for Computational Neuroscience, Berlin, Germany

## Abstract

Feedback is central to most forms of learning, and its reliability is therefore critical. Here, we investigated the effects of corrupted, and hence unreliable, feedback on perceptual inference. Within the framework of Bayesian inference, we hypothesised that corrupting feedback in a demanding perceptual task would compromise sensory information processing and bias inference towards prior information if available. These hypotheses were examined by a simulation and in two behavioural experiments with visual detection (experiment 1) and discrimination (experiment 2) tasks. Both experiments consisted of two sessions comprising intervention runs with either corrupted or uncorrupted (correct) feedback, and pre- and post-intervention tests to assess the effects of feedback. In the tests alone, additional prior beliefs were induced through predictive auditory cues to assess sustained effects of feedback on the balance between sensory evidence and prior beliefs. Both experiments and the simulation showed the hypothesised decrease in performance and increased reliance on prior beliefs after corrupted but not uncorrupted feedback. Exploratory analyses indicated reduced confidence regarding perceptual decisions during delivery of corrupted feedback. Our results suggest that corrupted feedback on perceptual decisions leads to sustained changes in perceptual inference, characterised by a shift from sensory likelihood to prior beliefs when those are accessible.

## Introduction

According to the Bayesian brain hypothesis, we make inferences about our environment by combining prior beliefs with current sensory evidence^1–3^. Within the Bayesian framework, perception results from the integration of probability distributions representing beliefs (‘prior’) and new sensory evidence (‘likelihood’). The resultant posterior distribution (‘posterior’) determines the perceptual outcome^4,5^. Moreover, the balance between the prior beliefs and the sensory evidence is thought to be dynamically adjusted depending on our estimates of their reliability, or precision^6,7^. Feedback helps us to arrive at these estimates by informing us of how well these two types of information predict outcomes. Here, we sought to understand how the reliability of feedback in a perceptual task influences the estimation of likelihood distributions and how this subsequently affects the balance between prior beliefs and sensory evidence once priors are available.

Previous work indeed suggests that reliability of feedback can influence perceptual inference. Corrupted feedback in an unrelated task leads to increased pattern perception in noisy images^8,9^. Moreover, increasing the uncertainty of feedback in a visuomotor task results in a stronger influence of prior beliefs on behaviour^10^. In the present study, we asked whether corrupted feedback on performance in a demanding perceptual task would subsequently lead to an increased reliance on prior beliefs in a situation where predictive information inducing such prior beliefs is available.

A key aspect of our investigation was to deliver corrupted feedback on perceptual performance when only the sensory evidence (and no additional predictive information) was available, and to then measure the effects in *subsequent* runs where two sources of information were provided on each trial – (1) a learned predictive cue inducing a prior belief and (2) the actual sensory evidence^11–13^. We reasoned that corrupting feedback on perceptual performance would lead to erroneous updating of likelihood distributions, rendering them more imprecise over time. Therefore, we hypothesised that as a result of such erroneous learning, corrupted feedback would subsequently lead to (1) a decrease in perceptual performance, and (2) when predictive information becomes available, to an increased reliance on prior beliefs^10,14^. Additionally, we collected confidence ratings on each trial in one of the experiments, to measure subjective awareness of changes in performance.

**Figure 1:**
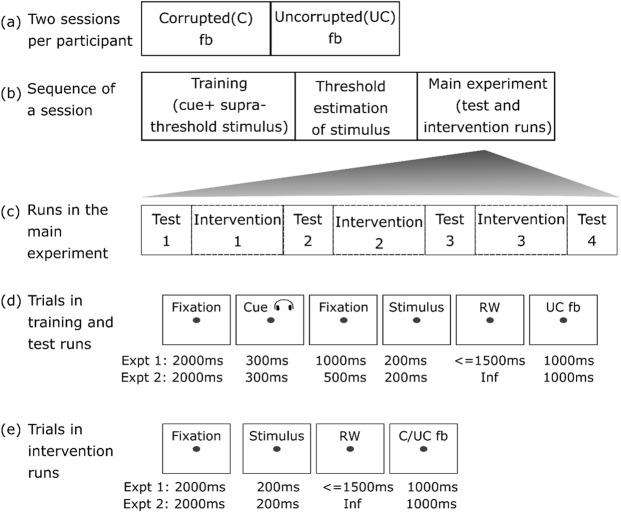
Design of experiments 1 and 2. (**a**) Each participant took part in two sessions, one with corrupted and one with uncorrupted feedback interventions. (**b**) Each session consisted of three parts: training, threshold estimation and the main experiment. (**c**) The main experiment comprised four test runs interleaved with three intervention runs. The intervention runs delivered either corrupted or uncorrupted feedback throughout a session. Timecourses of trials in (**d**) training and test runs, and (**e**) intervention runs.

We tested these two hypotheses by studying the effects of corrupted feedback in two behavioural experiments and through simulations of misclassification-induced learning. In the behavioural experiments, each participant received corrupted and uncorrupted feedback in two separate sessions on different days (Fig. 1a). Each session started with preliminary *training* runs to learn the priors and was followed by threshold estimation runs to set the perceptual threshold for the main experiment (Fig. 1b). This was then followed by the main experiment, comprising *intervention* runs, during which corrupted or uncorrupted feedback regarding the perceptual choice was given after each trial, and pre- and post-intervention *test* runs (Fig. 1c). Only during these test runs, but not during the interventions runs, was each visual stimulus preceded by a probabilistic auditory cue to induce a prior belief (Fig. 1d,e). This design allowed us to measure the effects of corrupted feedback both on visual task performance and on the participants’ reliance on experimentally induced prior beliefs (once predictive information to induce prior beliefs was available). Importantly, corrupted feedback was never presented in the runs with the auditory cues. Uncorrupted feedback was delivered in the test runs in order to reinforce the cue-stimulus association learnt in the training runs. Thus, corrupted feedback could only interfere with the updating of the likelihood representations, but not with the updating of the priors. Both experiments 1 and 2, as well as the simulation followed this experimental design.

**Figure 2:**
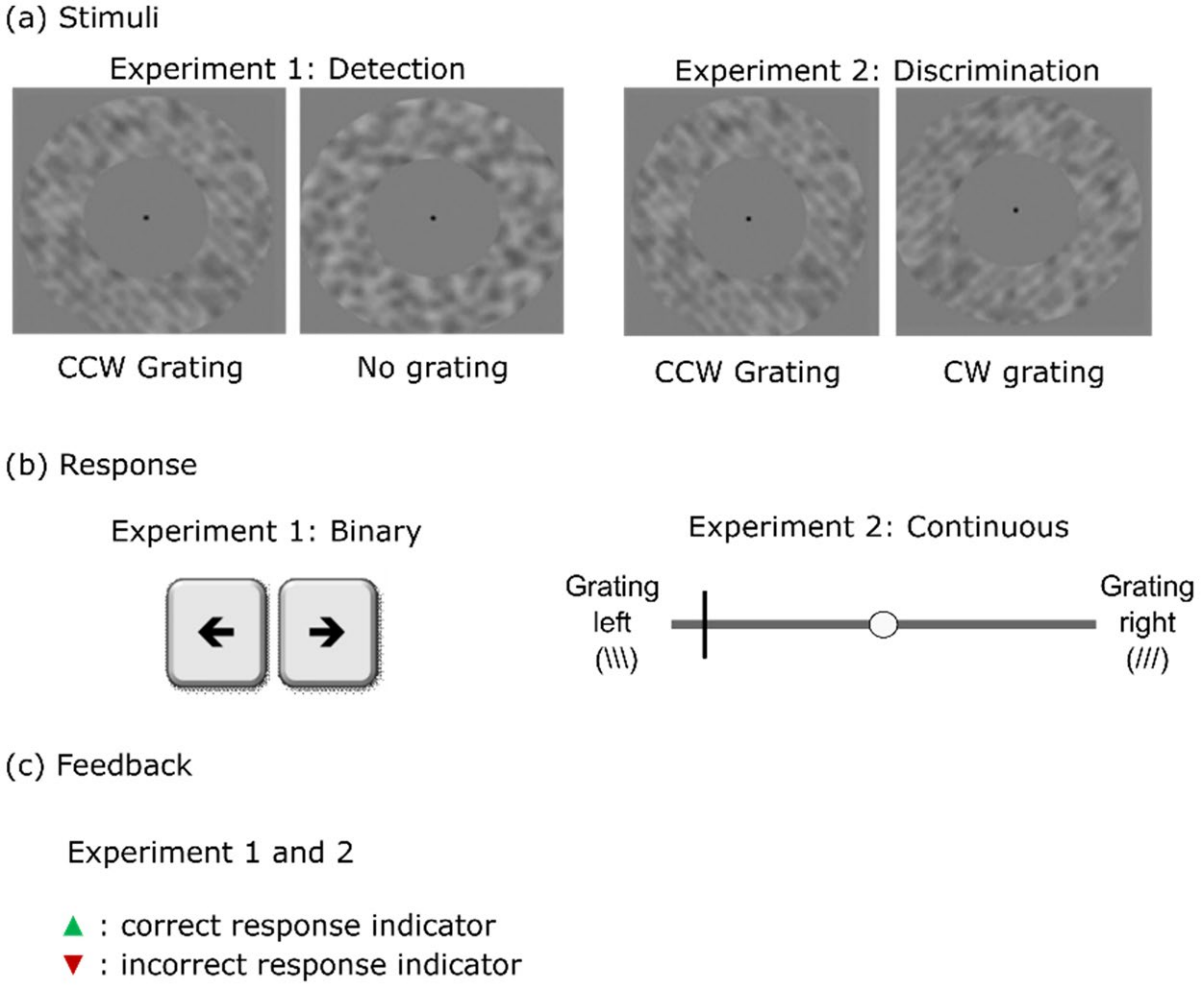
(**a**) Visual stimuli (CCW: counter-clockwise, CW: clockwise), (**b**) response modalities and (**c**) visual feedback used in experiments 1 and 2.

**Figure 3:**
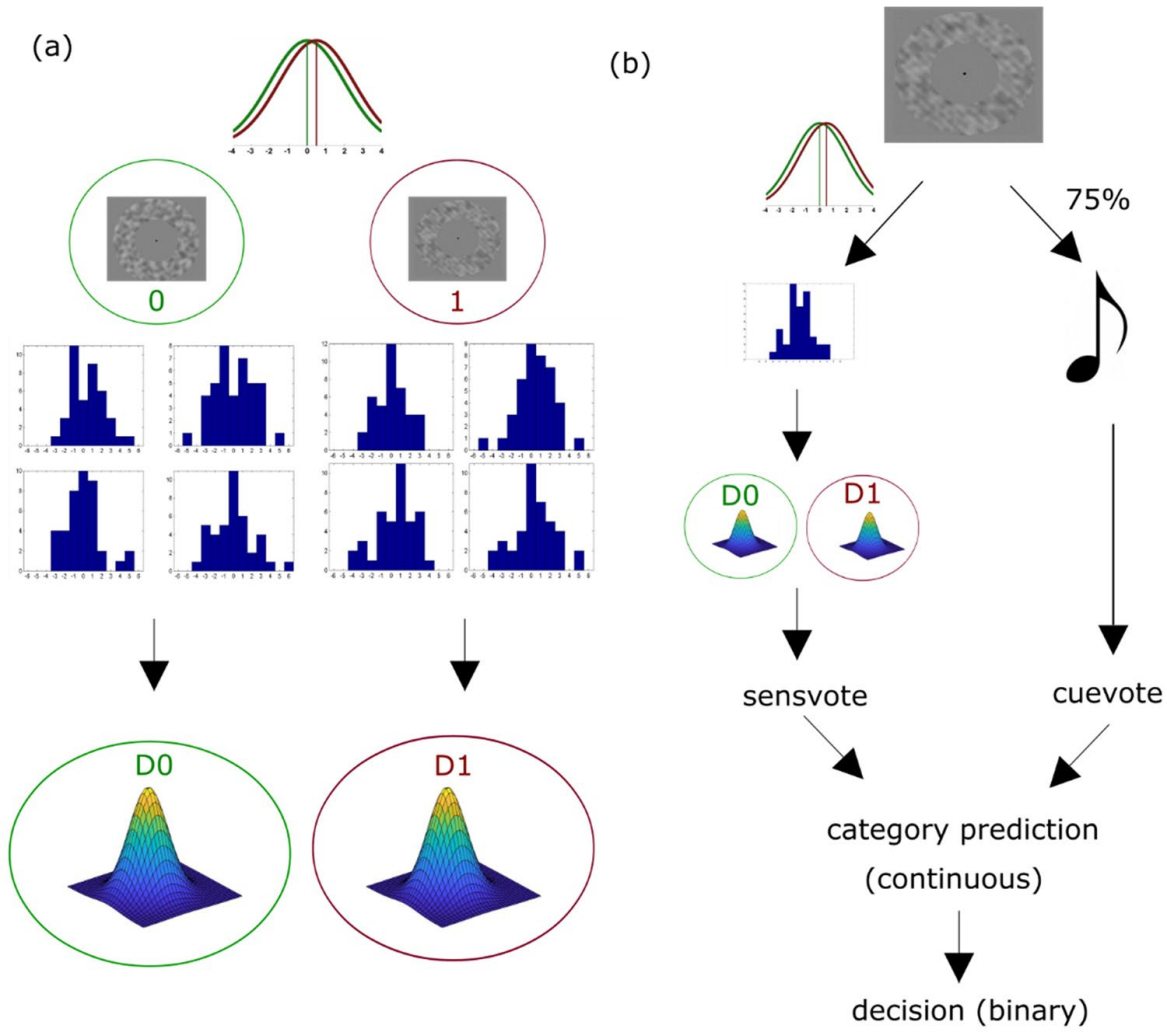
(**a**) Training of the sensory classifier in the simulation (training and intervention runs). Two normal-gamma distributions (D0 and D1) were fed with samples (histograms) from two normal distributions ‘0’ (“target absent”) and ‘1’ (“target present”). (**b**) Decision-making in the simulation (test runs). A vote of the sensory classifier about the presence of absence of target (sensvote) was obtained by comparing the likelihoods of the sensory data between D0 and D1. This vote was combined with cue information (cuevote) in a logistic function to make decisions (Equations 3–5, Materials and Methods).

Experiments 1 and 2 consisted of visual detection and discrimination tasks, respectively (Fig. 2a). To understand how impaired learning about the causes of sensory data (as induced by corrupted feedback) might subsequently lead to decreased perceptual performance and an enhanced reliance on learned priors, we simulated behaviour in an artificial observer. In the simulation, decision-making was implemented in the form of a high-level “decision classifier”, which combined the vote of a “sensory classifier” that evaluated the sensory data with the prior belief established by the cue (Fig. 3).

## Results

The present study tested two main hypotheses regarding the sustained effects of corrupted feedback delivered in a perceptual task in the absence of priors: that in subsequent runs without corrupted feedback (1) it deteriorates task performance and (2) in the presence of predictive information, it shifts choices towards prior beliefs. We investigated both hypotheses by means of simulations and based on empirical behavioural data gathered in experiments 1 and 2. 37 participants (6 male, ages 27.7 ± 5.9) took part in experiment 1, three of whom had to be excluded due to technical difficulties (i.e. final N = 34). 32 participants (7 male, ages 24.75 ± 3.6) took part in experiment 2. The mean time between the two sessions corresponding to corrupted and uncorrupted feedback (Fig. 1a) were 1.94 days (*SD* = 1.98 days) in experiment 1 and 1.7 days (*SD* = 1.7 days) in experiment 2. 1000 artificial subjects were created using the simulations.

### Corrupted feedback impairs performance

**Figure 4:**
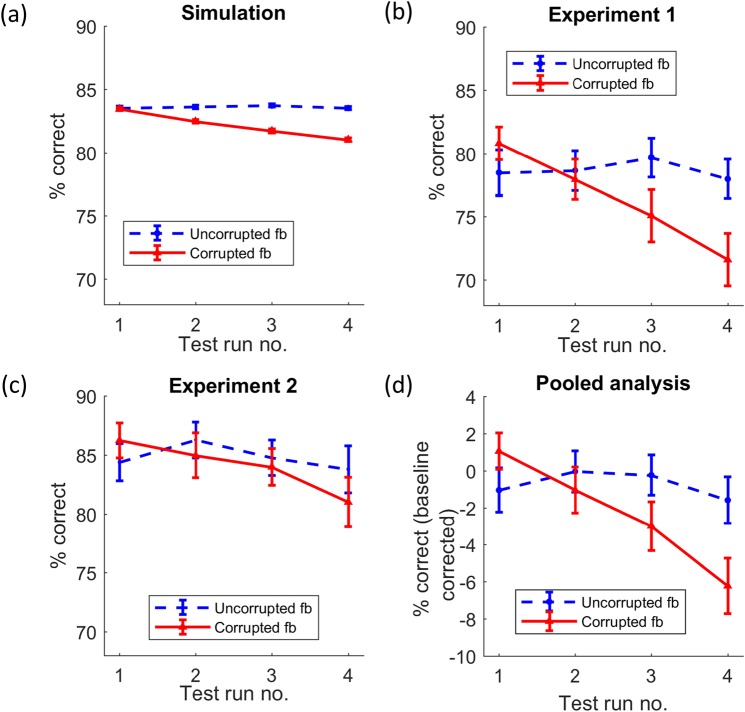
Changes in overall performance across time and feedback types in (**a**) the simulated data (n = 1000), (**b**) experiment 1 (n = 34), (**c**) experiment 2 (n = 32) and (**d**) the pooled data across experiments 1 and 2 (n = 66). The pooled data in (**d**) has been corrected for baseline performance differences between the two experiments. Errorbars indicate standard errors.

The critical analysis to assess the influence of corrupted feedback on performance was the interaction between the within-subject factors feedback type (corrupted, uncorrupted) and time (test runs 1 to 4). This was tested by means of a two-way repeated measures analysis of variance (RM-ANOVA). More specifically, using a linear ANOVA contrast we tested the hypothesis that the linear performance change across time was different between the corrupted and uncorrupted feedback conditions (henceforth referred to as *linear interaction effect*). This linear interaction effect was significant (*F*(1,999) = 92.42, *p* < 0.001, *η*_*p*_^2^ = 0.09) for the simulated data in line with our hypothesis (Fig. 4a). Post-hoc analyses showed that the observed interaction was based on a decrease in performance, indicated by the significantly negative slope across time in the corrupted feedback session (*M* = −0.8, *SE* = 0.07, *t*(999) = −12.32, *p* < 0.001) and a non-significant slope across time in the uncorrupted feedback session (*M* = 0.01, *SE* = 0.06, *t*(999) = 0.14, *p* = 0.89). Thus, the simulation attested to our hypothesis that corrupted feedback impairs performance in perceptual decision making.

Next, we assessed whether the behavioural data in experiments 1 and 2 would similarly show changes in performance in the corrupted feedback condition (Fig. 4b,c). In line with our hypothesis and the simulation, we found a significant linear interaction effect between the factors feedback type (fbtype) and test run number (time) in experiment 1 (*F*(1,29) = 7.93, *p* = 0.01, *η*_*p*_^2^ = 0.22), but not in experiment 2 (*F*(1,27) = 1.5, *p* = 0.23, *η*_*p*_^2^ = 0.05). However, the session-wise slopes for performance were negative in the corrupted feedback sessions in both the experiments (experiment 1: *M* = −3.05, *SE* = 0.66, *t*(33) = −4.64, *p* < 0.001; experiment 2: *M* = −1.67, *SE* = 0.74, *t*(31) = −2.24, *p* = 0.03), but not in the uncorrupted feedback sessions (experiment 1: *M* = −0.05, *SE* = 0.54, *t*(33) = −0.09, *p* = 0.93; experiment 2: *M* = −0.33, *SE* = 0.71, *t*(31) = −0.46, *p* = 0.65). To exclude effects due to the between subject factors (tone-stimulus association type, sequence of sessions) and covariate (number of days between sessions), these terms were included in the ANOVA tests. They did not show significant interactions with fbtype and time (all *p* > 0.27).

Since both experiments followed the same design and varied only in terms of the stimuli and the response modality (see Fig. 2 and Materials and Methods), we performed a post-hoc RM-ANOVA on the pooled dataset with *experiment number* (i.e., 1 or 2) as an additional between-subject factor. This analysis revealed a significant linear interaction effect between time and fbtype (*F*(1,57) = 8.63, *p = *0.005, *η*_*p*_^2^ = 0.13) (Fig. 4d), and like in the individual experiments, resulted from a decline in performance in the pooled dataset in corrupted feedback session (slope *M* = −2.38, *SE* = 0.5, *t*(65) = −4.78, *p* < 0.001), but not in the uncorrupted feedback condition (slope *M* = −0.18, *SE* = 0.44, *t*(65) = −0.42, *p* = 0.68). The three-way linear interaction between fbtype, time and experiment number was not significant (*F*(1,57) = 2.4, *p* = 0.13), indicating that the fbtype-by-time interaction was comparable across experiments.

In parallel with the performance changes in the test runs, the linear interaction effect between fbtype and time was significant in the intervention runs too (Fig. S1, hollow triangles and circles). Detailed results of the analyses performed on data from the intervention runs are presented in the Supplementary Results Section.

Taken together, these results show that corrupted feedback systematically impairs the accuracy of perceptual decision making.

### Corrupted feedback on perceptual decisions increases the influence of prior beliefs

**Figure 5:**
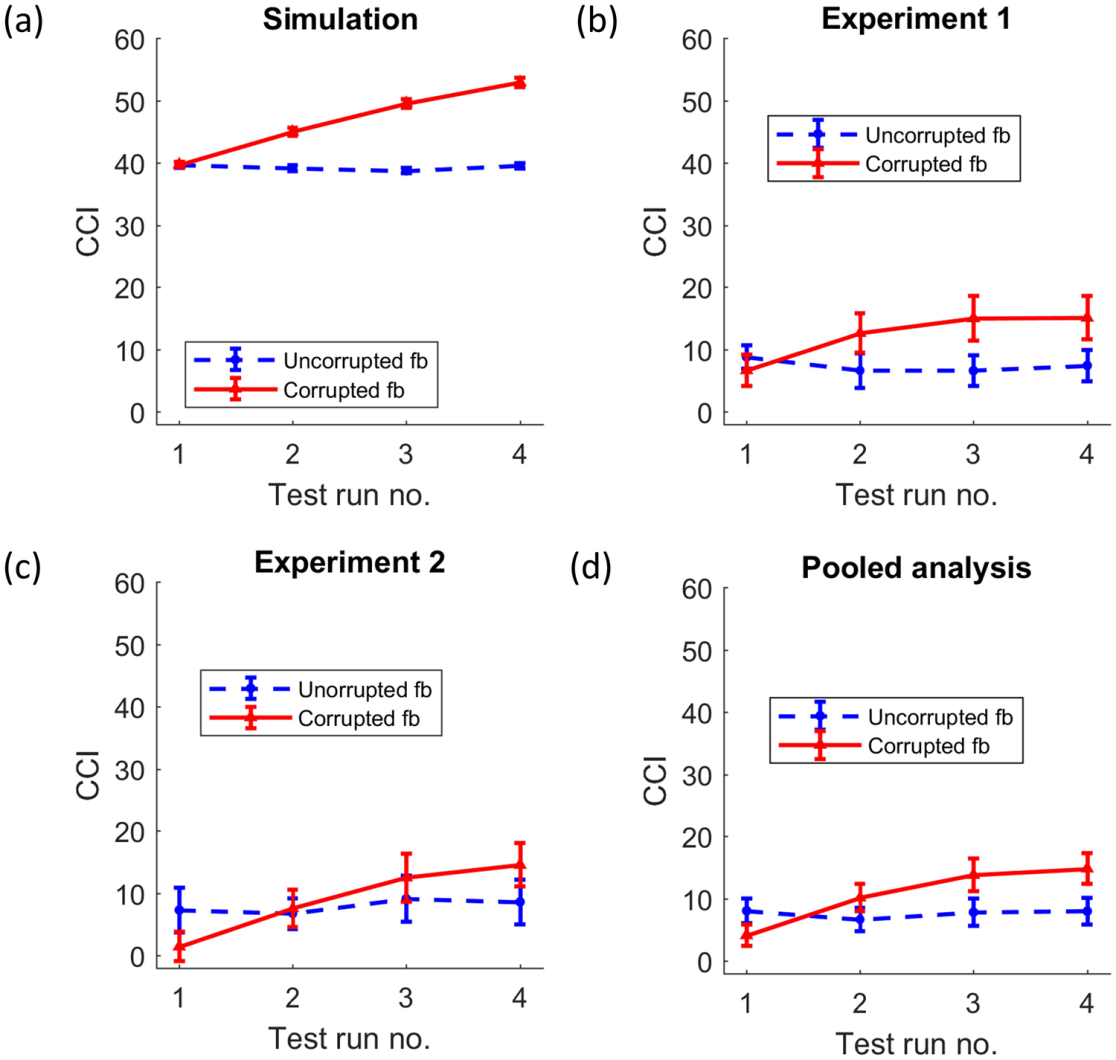
Changes in the cue congruence index (CCI) across time and feedback types in (**a**) the simulated data (n = 1000), (**b**) experiment 1 (n = 34), (**c**) experiment 2 (n = 32) and (**d**) the pooled data across experiments 1 and 2 (n = 66). Errorbars indicate standard errors.

Cue congruence indices (CCI) were computed for each test run (see Materials and Methods) in order to study the influence of learned prior beliefs on perceptual decisions after corrupted feedback interventions. Again, the critical analysis was the linear interaction between time and fbtype. As predicted, the simulation showed a significant linear interaction effect for CCI (Fig. 5a; *F*(1,999) = 166.7, *p* < 0.001, *η*_*p*_^2^ = 0.14). Further in line with our second hypothesis, CCI increased over time in the corrupted feedback session, resulting in a positive slope (*M* = 4.41, *SE* = 0.29, *t*(999) = 15.06, *p* < 0.001), and did not change in the uncorrupted feedback session, showing no change in slope (*M* = −0.08, *SE* = 0.2, *t*(999) = −0.39, *p* = 0.69).

The linear interaction effect between fbtype and time did not reach the significance threshold in experiment 1 (Fig. 5b; *F* (1, 29) = 2.81, *p* = 0.1, *η*_*p*_^2^ = 0.09), but it did so in experiment 2 (Fig. 5c; *F*(1, 27) = 4.51, *p* = 0.04, *η*_*p*_^*2*^ = 0.14). However, the session-wise slopes showed that there was an increase in the CCI across time in the corrupted feedback session both in experiment 1 (slope *M* = 2.76, *SE* = 1.14, *t*(33) = 2.42, *p* = 0.02) and experiment 2 (slope *M* = 4.42, *SE* = 1.43, *t*(31) = 3.09, *p* = 0.004), and that there was no change over time in the uncorrupted feedback session (experiment 1: *M* = −0.41, *SE* = 1.04, *t*(31) = −0.4, *p* = 0.69*;* experiment 2: *M* = 0.63, *SE* = 1.39, *t*(31) = 0.45, *p* = 0.65). None of the between-subject factors and covariates interacted significantly with fbtype and time (all p > 0.08).

Lastly, a post-hoc RM-ANOVA of the pooled data (from experiments 1 and 2) was performed with the additional between-subject factor *experiment number*. This analysis revealed a significant linear interaction between fbtype and time (Fig. 5d; *F*(1,57) = 6.76, *p* = 0.01, *η*_*p*_^*2*^ = 0.11), which was explained by a positive slope for corrupted (*M* = 3.57, *SE* = 0.91, *t*(65) = 3.93, *p* < 0.001) and a non-significant slope for uncorrupted (*M* = 0.09, *SE* = 0.86, *t*(65) = 0.11, *p* = 0.91) feedback. Our results thus show that corrupted feedback on perceptual decisions increases the reliance on priors once they are available.

### Corrupted feedback decreases confidence in responses during feedback delivery

**Figure 6:**
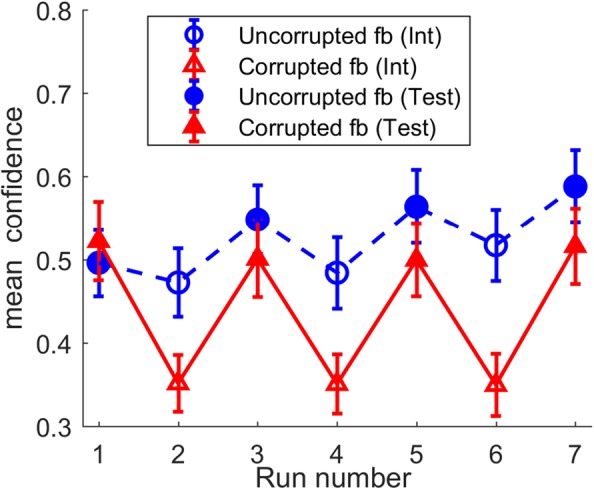
Changes in mean confidence across the test runs (filled triangles and circles), the intervention runs (unfilled triangles and circles) and the feedback types (solid and dashed lines) in experiment 2. Errorbars denote standard errors.

With the confidence ratings collected in experiment 2, we explored whether corrupted feedback would give rise to reduced confidence in perceptual decisions. Confidence was encoded as a decimal value in the interval [0.03, 1], where 0.03 and 1 were the lowest and highest possible ratings of confidence, respectively (see Materials and Methods). Similar to the earlier analyses, we first tested for a linear interaction between fbtype and time on the confidence ratings in the test runs. Contrary to the results from the objective measures (performance and CCI), no interaction was observed for the subjective measure of perception, i.e., confidence (*F* (1, 27) = 0.84, p = 0.37, η_p_^2^ = 0.03), although there was a slight increase in confidence across time in the uncorrupted feedback sessions (slope *M* = 0.03, *SE* = 0.01, *t*(31) = 3.12, *p* = 0.004) and not corrupted feedback sessions (slope *M* = −0.002, *SE* = 0.01, *t*(31) = −0.2, *p* = 0.84). However, as visible from Fig. 6, confidence ratings showed sharp drops of confidence during intervention runs in the corrupted feedback session (average confidence: *M* = 0.35, *SE* = 0.03), which relaxed to baseline in subsequent test runs (*M* = 0.51, *SE* = 0.04). A paired t-test of mean confidence in test runs and that in intervention runs showed that the drop in confidence was highly significant (*M* = −0.16, *SE* = 0.02, t(31) = −6.67, p < 0.001) in the corrupted feedback session. Although there was a slight decrease in mean confidence across intervention runs compared to test runs in the uncorrupted feedback session as well, the difference here was much smaller (*M* = −0.06, *SE* = 0.01, *t*(31) = −4.31, *p* < 0.001). Thus, while corrupted feedback has long-lasting effects on objective measures of perceptual inference that transfer to test runs, it exerts a short-term effect on the subjective measure of confidence, by reducing confidence only *during* the actual intervention.

### Other post-hoc tests

Subjective awareness of the feedback manipulation may have influenced task performance. To investigate the impact of such awareness on the main results (i.e., the decrease in performance and the increase in cue congruence), we compared the self-reported awareness of feedback manipulation, obtained during debriefing, with the differences in slopes between sessions (Fig. S2). The results revealed that greater awareness of manipulation did not enhance the effects. In fact, the differences between session-wise slopes for performance and cue congruence were smaller in magnitude in those participants who reported being aware of the external manipulation of feedback in the intervention runs, although this effect was observed only in experiment 1. The analysis steps and results are described in detail in the Supplementary Section.

A second post-hoc analysis investigated the influence of motivation on performance and cue congruence. This analysis was performed only for experiment 2, where motivation ratings were collected for each run. We tested for correlations of the slope difference for motivation with analogous differences in slopes of the main dependent variables (performance and CCI). However, we found no significant correlation and hence no evidence for a direct influence of motivation on the observed effects (Fig. S3). The methods and results of these analyses are described in more detail in the Supplementary Section.

A third post-hoc test investigated whether the initial position of the cursor had any influence on the responses in experiment 2. This was tested by first estimating the overall correlation coefficients between the two variables and then estimating systematic changes in them across time and fbtype. The two analyses did not show consistent relationships between the initial and final cursor positions (Fig. S4). Thus, the randomisation of the initial mouse position appeared not to bias the responses in experiment 2. The analysis is described in detail in the Supplementary Section.

## Discussion

In the present study, we investigated the influence of corrupted feedback on perceptual inference using detection and discrimination tasks in two independent experiments. We found that when corrupted feedback was delivered in a perceptual task in the absence of priors, (1) task performance deteriorated, and (2) perceptual inference was shifted towards learned priors, after corrupted feedback delivery stopped.

The decrease in performance was stronger in experiment 1 than in experiment 2, the latter showing a negative performance slope for the corrupted feedback session, but no significant linear interaction between fbtype and time. This difference between the two experiments could be due to several reasons. One possibility is that the baseline performance (test run 1) was higher in experiment 2, thereby reducing task difficulty, and consequently the disruptive effect of corrupted feedback. The task could have also been less perceptually demanding, since more time was given to make responses on the continuous scale in experiment 2. Finally, the effect of corrupted feedback may simply differ between detection and discrimination tasks. Different neural mechanisms have been proposed to underlie detection and discrimination, both in terms of the neurons encoding visual stimuli at lower levels^15^ and in the cognitive resources required to perform the tasks^16^. Discrimination has been suggested to be more demanding and to involve two subsets of neurons instead of one. However, we see the opposite effect in our results, thereby making this an unlikely explanation for the differences seen between experiments 1 and 2.

Previous studies have already shown trends in the direction of our observations – that corrupted feedback prevents learning^17,18^, changes the sensitivity to stimuli^19^ and induces false percepts even after delivery of such feedback stops^8,9^. Further, studies have also shown that a decrease in the precision of likelihood distributions can shift the inference towards priors^10,14^. In our experiments, since the prior (defined by a fixed cue-stimulus association) did not interact with corrupted feedback across the course of the experiment, the observed increase in cue-congruent responses was likely due to the decline in accuracy in the processing of sensory information. Importantly, if corrupted feedback would have been presented in the presence of predictive cues, the precisions of both the prior and the sensory evidence would likely have been affected, which may have reduced or even nullified the prior-congruent behaviour. Here, in order to delineate the influence of corrupted feedback on the processing of the bottom-up sensory evidence and to prevent a direct learning between cues and corrupted feedback, we kept the prior separate from the feedback manipulation.

Conceivably, non-perceptual mechanisms may have influenced our results. One such possibility is that the observed results could have stemmed from a mechanism akin to “learned helplessness”, resulting from the lack of control induced by corrupted feedback^8^. However, while lack of control is known to impair performance^20,21^, this mechanism cannot explain the increase in cue congruence (CCI). The shift in responses towards cues was not deliberate, since such a shift in response strategy would have given high CCIs (approaching the maximum value of 100, see Materials and Methods). On the other hand, in our experiments, the highest average CCI attained was 15 (Fig. 5b,c) – suggesting a slow and rather automatic shift in responses towards the prior.

In both our experiments, direct comparisons were made between sessions with corrupted and uncorrupted feedback. A possible explanation of our results could thus also be that the observed effects of corrupted feedback were due to the *absence* of uncorrupted feedback rather than the *presence* of corrupted feedback. Findings from previous studies make this interpretation unlikely, since learning can occur even in the absence of feedback at the 80% performance threshold^22,23^, while we found a decline in performance under corrupted feedback. However, to further investigate the differences between corrupted feedback on the one hand and absence of feedback on the other, future research should directly compare the effects of these two conditions on perceptual learning.

Interestingly, when investigating the influence of corrupted feedback on confidence in experiment 2, we found that confidence decreased in the intervention runs but that it was restored in the ensuing test runs. Thus, it appears that the *subjective* measure of perception (confidence) showed only short-term effects (i.e., within the intervention runs), whereas the *objective* measure of perception (performance) showed a more long-term effect extending to the ensuing test runs as well. Since confidence is an indicator of performance as well as metacognition^23–25^, it is possible that confidence mirrors performance. However, it must be noted that although the relative differences between sessions (corrupted–uncorrupted) are similar between performance and confidence, the actual events are slightly different: corrupted feedback *prevents an increase* of confidence whereas it *decreases* performance accuracy. The influence of corrupted feedback on performance and confidence should be tested in a future experiment where uncorrupted feedback is not delivered in the test runs, which might help to counteract the immediate restoration of performance and confidence.

We also performed post-hoc tests to identify potential confounds in our results due to participants’ awareness of the feedback manipulation, subjective motivation and response bias due to the cursor’s initial position (the last two tests only for experiment 2). Results revealed that the effects (differences in session-wise slopes between sessions) did not increase with increasing awareness of feedback manipulation. In fact, experiment 1 showed that higher awareness of feedback manipulation reduced the differences between sessions. Changes in motivation did not correlate with changes in performance and cue congruence either. Lastly, the initial position of the cursor on the response bar in each trial did not correlate with the responses. Thus, the changes in performance and cue congruence observed in the experiments were unlikely to have arisen from strategies deliberately adopted by participants or from differences in subjective motivation.

Recently, theories of Bayesian learning in the brain and its potential pathological aberrations have inspired cognitive models of psychiatric disorders such as schizophrenia^7,26,27^. It has been proposed that false inferences regarding the environmental causes of sensory input data might lead to an unstable representation of the environment, which would in turn appear unpredictable and potentially threatening. While this notion may account for a variety of cognitive and perceptual aberrations observed in schizophrenia, it cannot easily explain one of its key features, namely, the stability of delusional beliefs, which are typically resistant to contradictory evidence. Consistent with the clinical importance of fixed delusional beliefs, it has been shown experimentally that individuals with growing delusion proneness exhibit a stronger tendency to perceive ambiguous stimuli in a manner congruent with induced prior beliefs^28^. This might engender a cycle of impaired sensory processing and compensatory strengthening of delusional beliefs, which might in turn shape perception in a belief-congruent (delusional) manner. Our current results demonstrate that impairments in sensory learning (as induced by feedback corruption) may indeed engender an enhanced usage of prior beliefs in order to compensate for suboptimal sensory models.

Taken together, the simulations and the experiments detailed in this paper suggest that the delivery of unreliable feedback has a debilitating effect on performance and subsequently skews perception towards existing prior beliefs when the prior is held stable. While we cannot differentiate at the behavioural level whether these effects stem from changes in sensory processing in the visual cortex or due to changes in higher-level decision-making processes^18,29^, future research using neuroimaging techniques could investigate the neural processes underlying the effects of corrupted feedback on perceptual inference.

## Materials and Methods

### Behavioural experiments

The study was approved by the ethics committee at Charité - Universitätsmedizin Berlin, and informed consents were collected from all participants. All the methods were carried out in accordance with the relevant guidelines and regulations. As both experiments were very similar in experimental design, they are described together here, and distinctions are made wherever the methodology differed.

#### Stimuli

Images were constructed from an overlay of annular gratings and noise images (Fig. 2a). Annular gratings at an orientation of 45° counter-clockwise or clockwise (the latter only for experiment 2) were generated such that the spatial frequency of the gratings would be 0.87 cycles/degree, the inner diameter of stimuli 9.94° and the outer diameter 20.93°. Noise images were generated by performing spatial smoothing of a two-dimensional annular noisy patch of the same inner and outer diameters as that of the gratings. Next, based on a previous study with noisy gratings^23^, the grating and noise images were combined in the following manner for the main task runs (Fig. 1c):

1 $${\boldsymbol{I}}=0.5\,(1+{{w}}_{{s}}\cdot {\boldsymbol{G}}+{{w}}_{{n}}\cdot {\boldsymbol{N}})$$

where **G** and **N** were two-dimensional matrices consisting of the grating and smoothed noise images respectively, scaled to the interval [−0.5, 0.5], and **I** the resultant image matrix. Parameters *w*_*s*_ and *w*_*n*_ were signal and noise weights respectively. The parameter *w*_*n*_ was maintained at a constant value of 0.25 across subjects and sessions, and *w*_*s*_ was set based on the signal threshold *s* (in percent) estimated prior to the main task for each participant during each session as follows:

2 $${w}_{s}={w}_{n}\cdot \frac{\,s}{100-s}.$$

#### Cues

Auditory tones of high (1000 Hz) and low (300 Hz) frequencies adjusted for loudness served as cues, in line with previous studies that used audio-visual associative learning cues to study the influence of priors on behaviour^11,13,30^. On each trial, a cue tone was played for 300 ms, and after a brief interval (1000 ms in experiment 1, 500 ms in experiment 2), the visual stimulus was presented. The tones were probabilistically coupled to stimuli of one type in 75% of the trials and with stimuli of the other type in 25% of the trials. The type of cue-stimulus association (type 1: high tone/stim1 and low tone/stim2; type 2: high tone/stim2 and low tone/stim1) was constant for each participant across sessions, and this was balanced between participants. Participants were also instructed to pay attention to the tones when present and were told that these could be helpful. They were not informed as to *how* useful the cue would be and whether the cue-stimulus association would change over time. To understand subjective perception of cue-stimulus association better, participants rated the perceived co-occurrence of cues and stimuli at the end of runs. This data proved to be unrevealing and is omitted here for brevity.

#### Feedback

Trial-by-trial visual feedback was delivered at the centre of the screen, in line with previous studies that have used colour-coded or symbolic cues^31–33^. An upward-pointing green triangle indicated a correct response and a downward-pointing red triangle indicated an incorrect response (equilateral triangles with 0.78° edges, see Fig. 2c). In runs with corrupted feedback, the presentation of the red/green triangles was pseudo-randomised, such that in half of the trials of each stimulus type, the feedback delivered was faulty.

#### Experimental procedure

The task was implemented using PsychToolbox 3.0.11 (psychtoolbox.org) on a computer screen (resolution: 1280 × 960 pixels, refresh rate: 60 Hz), placed 46 cm away from the chinrest, where the participant was positioned. Participants were instructed to fixate at the centre of the screen throughout the experiment, where a black dot (radius: 0.34° visual angle) was presented at all times *except* during feedback delivery, when the feedback (Fig. 2c) replaced the dot. In experiment 1, the participants’ task was to report the presence or absence of gratings using the left and right arrow keys on a standard German keyboard. In experiment 2, the task was to report both the perceived orientation of gratings and the confidence about the response on a linear visual analogue scale using a single mouse-click (Fig. 2b, right). The left and right halves of this scale corresponded to the perception of counter-clockwise and clockwise gratings, respectively. The distance from the centre (white circle) indicated confidence, i.e., responses closer to the left and right tips of the scale indicated high levels of confidence about the respective percept, and those near the centre indicated low levels of confidence. It was not possible to click at the centre of the response bar, forcing participants to indicate a decision about the orientation to proceed. To minimise the effects of fatigue or laziness on confidence ratings, the initial position of the cursor was random on each trial. To reduce reporting errors in confidence, time restriction was not imposed in experiment 2.

#### Task design

There were two experimental sessions for each participant (Fig. 1a). The only difference between the two sessions was the presence of *corrupted* feedback in the intervention runs of one session and *uncorrupted* feedback in corresponding intervention runs of the other session.

Each session lasted for about two hours, including the time taken for the breaks, task instructions, training, threshold estimation and debriefing. The main experiment (four test and three intervention runs, Fig. 1c) lasted for approximately 70 minutes, with each test run lasting for approximately 6 minutes, and each intervention run approximately 9 minutes. Participants were encouraged to take short breaks between runs in order to minimise the effects of fatigue on behaviour.

The order of sessions was counter-balanced across participants. Each session consisted of three parts: training, threshold estimation and the main experiment (Fig. 1b). These parts are described below:

1. *Training*. In this part, an association was induced between auditory cues and visual stimuli. To facilitate this associative learning, supra-threshold stimuli (12% signal) were presented, and uncorrupted feedback was given. There were three runs in the training phase, and each run consisted of 48 trials. The timecourse of a trial in the training phase was as in Fig. 1d.
2. *Threshold estimation*. A staircase procedure was used to determine the percentage of signal (grating) required to attain a performance level of 80% correct responses. To estimate the signal threshold (*s* in Equation 2), a 2-down-1-up staircase procedure with two phases was performed before each session with a step-size down/step-size ratio of 0.5548^23,34^. The first phase was to determine the approximate signal threshold and had larger step-sizes (1% signal up, 0.5548% signal down). The second phase started at the threshold estimated by the first staircase and had smaller step-sizes (0.5% signal up, 0.2774% signal down). No auditory cues were presented during the staircase, but uncorrupted feedback was provided. In experiment 1, the first and the second phases of the staircase proceeded until a certain number of reversals were attained (8 and 10 for phases 1 and 2, respectively), or 80 trials were completed. The signal threshold was determined based on the signal levels at which the last 4 reversals occurred in the second phase. Experiment 1 showed that six reversals were sufficient to arrive at the threshold signal. We therefore confined threshold estimation to six reversals in experiment 2, while keeping everything else the same as in experiment 1.
3. *Main experiment*. The main experiment in both the sessions comprised seven runs in total (Fig. 1c). In all of them, visual stimuli were presented at the 80% performance threshold determined in the previous step. The test runs served to probe the sustained behavioural changes resulting from the feedback manipulation. Each test run consisted of 64 trials. The trials here were similar to the training runs, comprising predictive cues and uncorrupted feedback (Fig. 1d). In the intervention runs, either corrupted (50% correct) or uncorrupted feedback (100% correct) was delivered. Feedback reliability was the same across intervention runs within a session. Each intervention run consisted of 128 trials (Fig. 1e). In experiment 2, at the end of each run, participants were asked to rate their motivation on a scale from 0 to 100.

#### Debriefing

At the end of the second session in both the experiments, participants were asked to fill a short questionnaire, which consisted of questions about their awareness of having received corrupted feedback. For more details, please see the Supplementary Methods Section.

#### Eyetracking

A video-based eye-tracker (Cambridge Research Systems, UK; sampling rate: 250 Hz; spatial accuracy: 0.05°) was used to monitor fixation throughout the experiment. A region of interest with radius 15 mm (1.87°) was defined around the centre of the screen. If the detected gaze was outside this region, the trial would not start, and as a cautionary note to the participant, the fixation dot (●) would switch to a ring (○) of the same radius until gaze was returned to the fixation area. After stable fixation for 700 ms, another 300 ms interval followed, after which the auditory cue or visual stimulus was presented, depending on the run type. Fixation was monitored during the presentation of the visual stimuli as well in the test runs. In case the fixation was broken, (i) stimuli disappeared and (ii) the fixation dot was replaced by a ring (○) at the centre, like the fixation check at the onset of each trial.

### Simulation

The simulation closely resembled the structure of the behavioural experiments, comprising separate test and intervention runs (Fig. 1c). In the intervention runs, the observer learnt to discriminate between the two stimulus types (“target-present” and “target-absent”) based on either corrupted or uncorrupted feedback. In the test runs, the simulated observer was likewise provided with probabilistic cues (co-occurrence in 75% of trials).

#### Procedure

The sensory data were modeled in the form of normal distributions with variance 4 and a mean value of either 0 (N0, “target absent”) or 0.5 (N1, “target present”). Variance and mean values were chosen such as to match the target performance (approximately 80% correct) of the staircase procedure in the behavioural experiment.

Training of the observer’s sensory classifier was based on two normal-gamma distributions (D0 and D1) that were used to learn mean value and variance of the two classes represented by the distributions N0 and N1. A normal-gamma distribution is a four-parameter distribution, which represents a probabilistic estimate of the moments of a normal distribution and is updated with new samples from this normal distribution (i.e., it is a conjugate prior for normal distributions with unknown mean and variance in Bayesian learning). This means that in the hypothetical case of n → ∞ correct observations (infinite samples from the true underlying normal distribution), the normal-gamma distribution would represent a certain estimate of the underlying normal distribution, and the posterior predictive distribution would then converge to the true distribution. The training procedure is shown schematically in Fig. 3a.

The likelihood that the new samples belong to one of the stimulus classes represented in the respective normal-gamma (D0 or D1) can be evaluated with the respective posterior predictive distributions (obtained by integrating over the normal-gamma distribution), each of which took the form of a student t-distribution. Hence, the likelihood of the data, given the predictive distribution of the normal-gamma, is a measure of the probability that the data is drawn from the normal distribution (N0 or N1) represented by the respective normal-gamma distribution. Here, the observer collected 40 samples from the normal distribution in each trial. Next, the vote of the sensory classifier (called *sensvote* here) was obtained by subtracting the log likelihood of the data given hypothesis 0 (samples are drawn from the normal distribution N0 represented in D0) from the log likelihood of the data given hypothesis 1 (samples are drawn from the normal distribution N1 represented in D1). Mathematically, this can be represented as follows:

3 $$sensvote=\,ln(p(X|D1))-\,ln(p(X|D0))$$

The sensory vote thus obtained is a measure of how much more likely the sensory data originates from the “target present” distribution N1 as compared to the “target absent” distribution N0. Thus, *sensvote* is a single value obtained for each trial, without an associated distribution or precision. Similarly, a trialwise *cuevote* was obtained as binary values 0 or 1, encoding “target absent” and “target present”, respectively. To identify their individual contributions to the actual stimulus (i.e., target absent/present), the regression coefficients corresponding to sensvote and cuevote (β_s_ and β_c_, respectively) were obtained by fitting these terms to the *actual* stimulus category using logistic regression. This is because at this stage, the simulated observer learns from uncorrupted feedback providing information about true stimulus categories, analogous to learning from uncorrupted feedback in the test runs of the behavioural experiments. The estimated regression coefficients are in theory comparable to precisions (or inverse variances) of distributions corresponding to different sources of information. Next, to estimate the behavioural outcome, the decision classifier took the form of a logistic regression with sensvote and cuevote as predictors, and then converted the predictions to binary decisions (Equations 4–5).

4 $$prediction=1+\frac{1}{1+\exp (c+\beta s\ast sensvote+\beta c\ast cuevote)}$$

5 $$\begin{array}{llll}{decision}\,= & 0\,{prediction} &  <  & 0.5\\  & 1\,{prediction} & \ge  & 0.5\end{array}$$

Predictions below and above 0.5 were assigned to the categories “target absent” and “target present”, respectively. The constant c was estimated using maximum likelihood optimisation (implemented in the fitglm routine of the Matlab Statistics and Machine Learning Toolbox) and was included to improve the flexibility of the model in case of unequal apriori probabilities of stimuli. The decision-making procedure is shown schematically in Fig. 3b.

#### Implementation

To mimic the performance of human observers at baseline, the sensory classifiers (distributions D0 and D1) were pre-trained with 20 samples of “stimuli” (N0 and N1). This was followed by alternating test and intervention run simulations similar to the behavioural experiments (Fig. 1c). In the test runs, stimuli were classified by the decision classifier based on sensory data and the cue information as described above. As the goal of the simulation was specifically to investigate the effect of learning from corrupted feedback, the sensory classifiers were not updated in the test runs. In the intervention runs, if the feedback was uncorrupted, the sensory classifiers (distributions D0 and D1) were updated with samples from distributions N0 and N1, respectively. However, in the corrupted feedback condition, each distribution (D0 and D1) was trained with N0 in one half of the trials and N1 in the other half, i.e., half of the virtual stimuli were mislabelled (analogous to faulty feedback). 1000 iterations were performed each with uncorrupted and corrupted feedback interventions. These were taken to be 1000 artificial “subjects” in data analysis.

We note that the simulation-based values for performance and CCI cannot be interpreted in absolute terms as these depend on arbitrary simulation parameters representing the initial moments of the two stimulus distributions. Thus, only *changes* in performance and CCI can be inferred from the simulation.

### Data analysis

#### Collected behavioural data

The collected responses were binary in experiment 1 and continuous in experiment 2 (Fig. 2b). On each trial in experiment 2, the response was stored as a decimal value within the interval [−1, 1], where the sign (−/+) indicated whether the grating was perceived to be tilted counter-clockwise or clockwise, and the absolute value indicated confidence. It was not possible to select responses in the interval [−0.03, 0.03] (see *Experimental Procedure* above).

#### Dependent variables

Two dependent variables were computed for each test run to test the main hypotheses: (1) the percentage of correct responses and (2) cue congruence index (CCI). Due to the correlation between stimuli and cues (co-occurrence in 75% of the trials), a decrease in performance would be paralleled by a decrease in cue congruence. To get a measure of cue congruence that does not depend on this performance-related change, we computed the percentage of correct responses (CR) separately in cue-congruent (CC) and cue-incongruent (CI) trials in a run, and then defined the cue congruence index (CCI) as a difference between them:

6 $${CCI}={C}{{R}}_{{CC}}-{C}{{R}}_{{CI}}$$

Thus, cue congruence or CCI increases if the performance in CC trials increases *relative* to that of CI trials. The upper bound for CCI is 100 and occurs when cue-congruent responses were made on each trial. A CCI value of 0 would indicate that the cue had no influence on responses.

Next, in order to understand the overall changes in performance and cue congruence within a session, we fitted linear functions across runs for each dependent variable and session, resulting in two slopes each for performance and cue congruence per participant.

Lastly, to study changes in subjective ratings of confidence in experiment 2, mean confidence was computed for each run.

Since responses in both the behavioural experiments were unspeeded (participants were instructed to be as accurate as possible) and since experiment 2 used a continuous response scale in which the location of a bar had to be accurately adjusted (to obtain confidence ratings), we focused on response accuracy and did not analyse reaction times.

#### Statistical analysis

Our hypotheses were tested by means of separate two-way RM-ANOVAs on the run-wise estimates of the two main dependent variables (performance and CCI). Fbtype and time (4 levels) were included as within-subject factors and the linear interaction between them studied using ANOVA contrasts with linear weights. While analysing experimental data, there were additional between subject factors: (1) sequence of sessions (a binary value indicating whether corrupted feedback was delivered in the first or second session), (2) cue-stimulus association (types 1 or 2, indicating different combinations of cues and stimuli) and (3) experiment number (only for the post-hoc pooled analyses). In addition, the duration between the sessions was used as a between-subject covariate. In addition, we performed one-sample t-tests of the session-wise slopes separately for corrupted and uncorrupted feedback sessions to understand the direction of change (positive and negative slopes to indicate linear increases and decreases across time, respectively). In experiment 2, additionally, the changes in mean confidence were studied using the same analysis.

#### Software

IBM SPSS Statistics 23 and MATLAB R2013b were used for all statistical analyses and simulation. The simulation additionally used functions from the Statistics and Machine Learning Toolbox.

## Supplementary information

Sustained effects of corrupted feedback on perceptual inference

## Data Availability

The datasets and simulations used in the current study are available from the corresponding author on reasonable request.
