# Supplementary material for: Sustained effects of corrupted feedback on perceptual inference
Source: Sci Rep. 2019 Apr 2;9:5537. doi: 10.1038/s41598-019-41954-z (PMC6445092; doi:10.1038/s41598-019-41954-z)
Supplement: Supplementary file 1 — Sustained effects of corrupted feedback on perceptual inference [file 41598_2019_41954_MOESM1_ESM.pdf]

Sustained effects of corrupted feedback on perceptual inference

Varrier RS[1,2], Stuke H[1], Guggenmos M[1§] and Sterzer P[1,2§]

[1] Department of Psychiatry and Psychotherapy,

Charité – Universitätsmedizin Berlin, Germany

[2] Bernstein Center for Computational Neuroscience, Berlin, Germany

[§]equal contribution

Supplementary Information

Supplementary methods: post-hoc analyses

We performed additional post-hoc analyses to test for relationships between the main effects of interest (decrease in performance and increase in CCI) and two subjective factors: (1) awareness of feedback manipulation and (2) motivation ratings. To this aim, the two effects of interest were quantified as slope differences (i.e., difference between slopes of corrupted and uncorrupted feedback sessions) for performance and CCI.

Awareness of the feedback manipulation

At the end of the second session in both the experiments, participants were asked to fill a short questionnaire, which consisted of questions about their awareness of having received corrupted feedback. The relevant questions are written below (verbatim). Questions 3 and 4 were added to the questionnaire after experiment 1, to get a more quantitative estimate of feedback manipulation awareness.

1. How did you find the feedback (helpful/ confusing/ no difference etc.)?
    a. On Day 1:
    b. On Day 2:
2. Did you notice anything odd about the feedback? If so, on which session/ day did you notice it?
3. How reliable (correct) was the feedback? (0=absolutely not reliable (correct), 100=totally reliable (correct))
    a. On day 1: 0% --100%
    b. On day 2: 0% -- 100%
4. During the experiment, did you think that the feedback was manipulated? Please circle your response
    a. On day 1: Definitely yes/ highly likely / maybe / highly unlikely/ definitely not
    b. On day 2: Definitely yes/ highly likely / maybe / highly unlikely/ definitely not

Based on the answers to the aforementioned questions, awareness of the corrupted feedback intervention was converted to the scalar values 0 (completely unaware, always trusted feedback), 0.5 (partially aware, noticed some oddity in feedback), 1 (completely aware, realized that the delivered feedback was sometimes faulty). In both experiments, Spearman's rank correlation coefficients were computed between the awareness of feedback manipulation and slope difference for each dependent variable.

Motivation ratings

After each run in the main experiment (Figure 1c) in experiment 2, participants were asked to rate their motivation on a scale from 0 to 100 (0% = not motivated at all, 100% = fully motivated). These ratings were fitted across test runs with linear functions, and slopes were computed for each session. In the final step, a *slope difference* was computed between the corrupted and uncorrupted feedback sessions. Two correlation analyses were performed with this slope difference, namely, with analogous slope differences of performance and CCI, using Pearson's correlation.

Supplementary results

Corrupted feedback impairs performance in the intervention runs

Similar to the analyses performed on the test run data, the influence of corrupted feedback on performance in the intervention runs of each experiment was studied using linear ANOVA contrasts with fbtype and time (intervention runs 1 to 3) as the within-subject factors of interest. The sequence of sessions and the number of days between sessions were included as the between-subject factor and covariate, respectively. Performance changes across intervention runs were similar to those in the test runs, i.e., there was a linear interaction between the factors fbtype and time (experiment 1: F(1,31) = 7.67, p = 0.01, $\eta_p^2 = 0.2$; experiment 2: F(1,29) = 4.82, p = 0.04, $\eta_p^2 = 0.14$), and resulted from a selective decrease in performance as evidenced by the significant negative slopes in the corrupted feedback sessions (experiment 1: *M* = –1.81, *SE* = 0.79, *t*(33) = –2.29, *p* = 0.03; experiment 2: *M* = –1.62, *SE* = 0.7, *t*(31) = –2.33, *p* = 0.03), but not in the uncorrupted feedback sessions (experiment 1: *M* = 0.77, *SE* = 0.56, *t*(33) = 1.37, *p* = 0.18; experiment 2: *M* = 1.37, *SE* = 0.8, *t*(31) = 1.72, *p* = 0.1). In both experiments, three-way interactions of fbtype and time with the between subject factor and covariate were not significant (all p > 0.23). The performance changes across test and intervention runs for each experiment is illustrated in Figure S1.

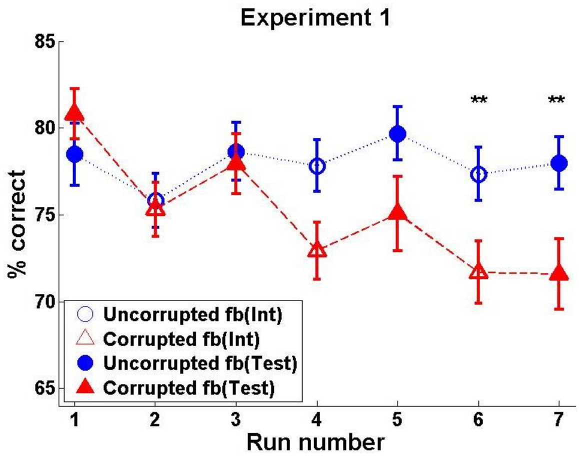

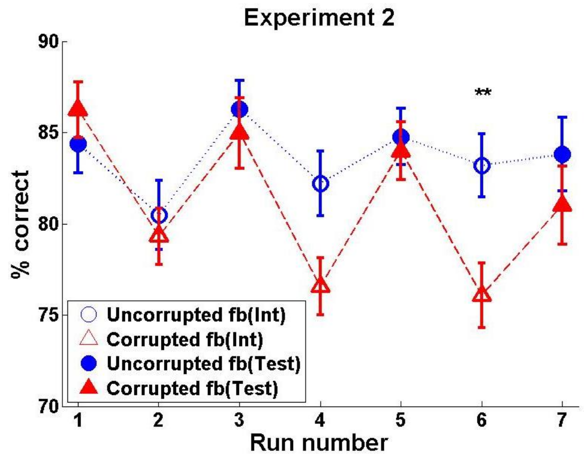

Figure S1: Changes in performance across runs on the corrupted (red, dashed lines) and uncorrupted feedback (blue, dotted lines) sessions in experiments 1(upper row) and 2 (lower row). Test runs (points 1,3,5,7 on the X-axis, filled triangle/circle) consisted of cued trials, and uncorrupted feedback was provided on both sessions. Intervention runs (at points 2, 4, 6 on the X-axis, unfilled triangles/circles) consisted of uncued trials, and provided corrupted or uncorrupted feedback. The asterisks (**) at a timepoint on the X-axis indicate that there was a significant difference ($p < 0.01$, paired t-test) between the corrupted and uncorrupted feedback sessions at that timepoint (run number). Errorbars denote standard error.

Results from the post-hoc tests

Higher awareness of feedback manipulation does not increase the observed changes in performance and CCI

We investigated whether post-experimental report of feedback-manipulation awareness could explain away the observed effects of corrupted feedback on performance and CCI. This was tested by correlating the variables encoding the awareness of feedback manipulation and slope differences of performance and CCI between test runs of corrupted and uncorrupted feedback sessions. The relationship between slope differences and feedback-manipulation awareness for each experiment and dependent variable are plotted in supplementary Figure S2.

In experiment 1, 23.53% of participants were completely unaware of the feedback manipulation (score 0), 32.35% were partially aware (score 0.5) and 44.12% were completely aware of the manipulation (score 1) at the end of the two sessions. Slope difference correlated (Spearman's rank correlation) with the awareness of feedback manipulation for both performance ($r = 0.37$, $p = 0.03$. supplementary Fig.S4, left upper) and CCI ($r = –0.35$, $p = 0.04$. supplementary Figure S2, left lower).

In experiment 2, 9.38% of participants were completely unaware of the manipulation (score 0), 43.75% were partially aware (score 0.5) and 46.88% were completely aware of the manipulation (score 1). Here, rated awareness of feedback manipulation did not correlate

significantly with performance (r = 0.03, p = 0.89, Figure S2, right upper) or CCI (r = –0.18, p = 0.31, Figure S2, right lower).

Thus, the performance and cue congruence effects, defined by differences in slopes between sessions could be diminished by a higher awareness of external manipulation of feedback. However, this effect was not consistent across the experiments. More importantly, the effects did not *increase* with increasing awareness of feedback manipulation, suggesting that the reported effects (decrease in performance, increase in CCI) were not the result of deliberate manipulation by participants.

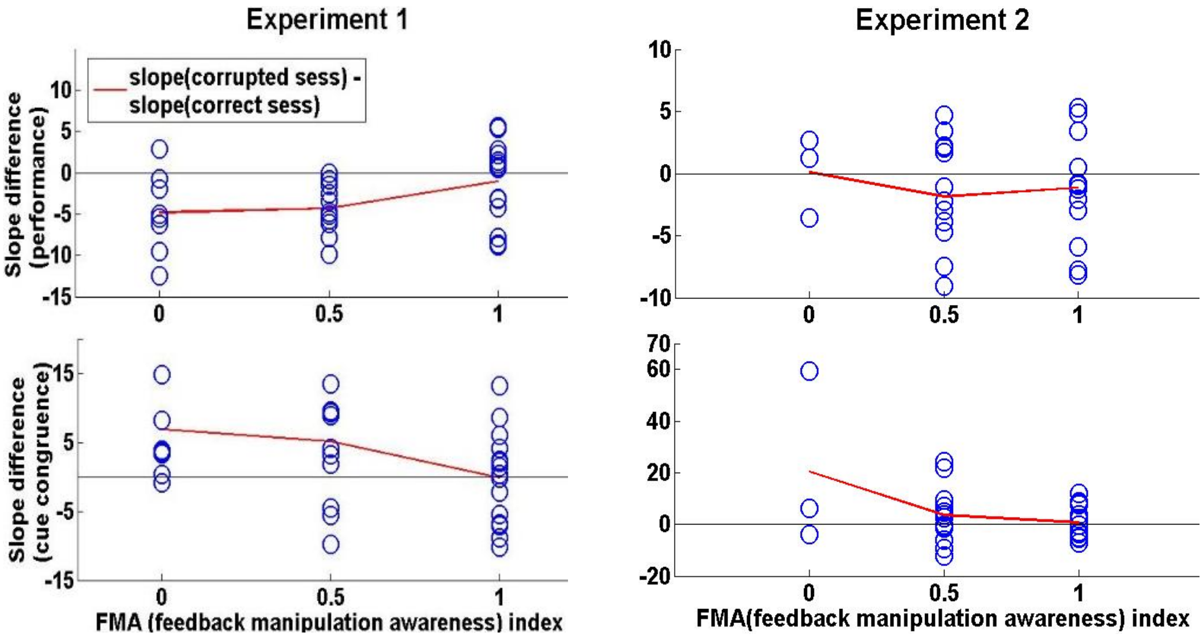

Figure S2: Relationship between the awareness of feedback manipulation sizes of the effect (computed as slope differences between corrupted and uncorrupted (correct) feedback sessions, for performance (upper left and right) and cue congruence (lower left and right) in experiments 1(left) and 2 (right). In all plots, circles represent individual participants, and the red lines in each plot connect the mean slope differences at each level of awareness (FMA index).

Motivation cannot explain the observed changes in performance and CCI

Run-wise motivation ratings were collected only in experiment 2. In general, motivation decreased across time, observed by means of a significant, negative slope of self-rated motivation across time and pooled across session types (mean difference = –2.08, p = 0.02, one-tailed t-test). Importantly, there was no significant difference in the slopes of motivation ratings between feedback types (mean difference = 0.5, p =0.7, paired t-test).

We next investigated the correlation of self-rated motivation with the dependent variables in experiment 2. Differences between session-wise slopes of motivation did not correlate with similar differences in performance (r = 0.05, p = 0.79). Similarly, the slope

difference for CC also did not correlate with motivation (r = –0.12, p = 0.52). Thus, our reported behavioural effects are unlikely to result directly result from changes in motivation. The results are summarised in Figure S3.

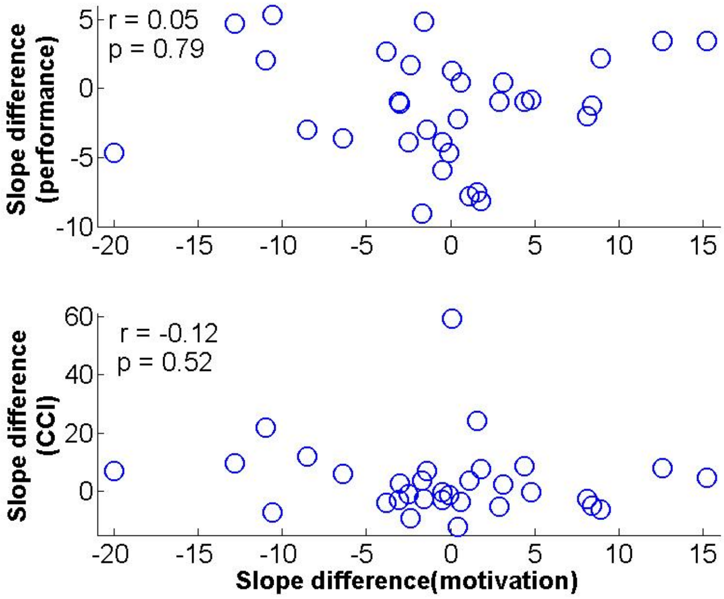

Figure S3: Slope differences for motivation (X-axis) plotted against performances (upper row) and cue congruence (lower row). Correlations coefficients (r) and their respective statistical significances (p) for each dependent variable is indicated next to the respective plots. In both plots, each circle represents a participant.

Initial cursor position does not influence responses

To test if the initial cursor position (randomly set in each trial, see Materials and Methods) could have had biased responses in experiment 2, , the correlation between the initial cursor position and the final cursor position (chosen response) was examined in two ways in the test runs. In the first part, correlation was computed after pooling data across fbtype and time. This would give giving one correlation coefficient (r) per participant. A Fisher z-transformation was then done on the coefficients (Z_r), and then a second-level analysis of the transformed coefficients was performed by means of a one-sample t-test. Z_r was not significantly different from zero (M= 0.02, p = 0.15), revealing that there was no significant correlation between the initial positions of the cursor and the participants' responses. To further investigate if such a correlation could have developed in the later runs as a result of fatigue or as a result of corrupted feedback, we computed z-transformed correlation coefficients (Z_r) for each run, and then performed a two-way RM-ANOVA using time and fbtype as the within-subject factors and using the same between-subject factors and covariates as in the analyses of the main dependent variables. Neither the main effect of time (F(1, 27) = 0.95, p = 0.34, η_p^2 = 0.03) nor the linear interaction between fbtype and time (F (1, 27) = 0.38,

$p = 0.54$, $\eta_p^2 = 0.01$) were significant (Figure S4). Thus, the randomisation of the initial cursor position did not bias participants' responses.

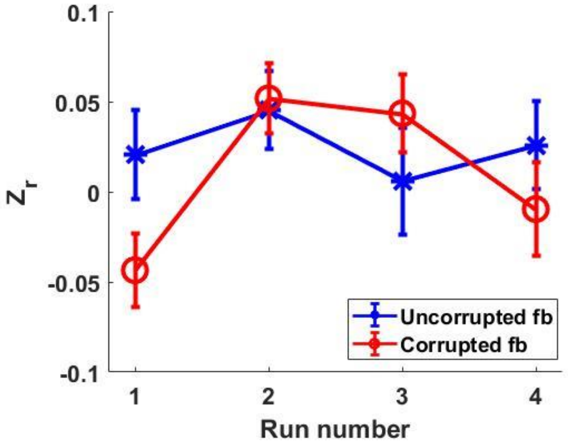

Figure S4: Changes in the Z-transformed correlation coefficient (Z_r, Y-axis) across time (X-axis) in the test runs of the corrupted (red, dashed lines) and uncorrupted feedback (blue, dotted lines) sessions in experiment 2. Errorbars denote standard error.
